# Low-Salt Intake during Mating or Gestation in Rats Is Associated with Low Birth and Survival Rates of Babies

**DOI:** 10.1155/2014/212089

**Published:** 2014-08-14

**Authors:** Ranna Chou, Anna Hara, DongDong Du, Namiko Shimizu, Hiroe Sakuyama, Yoshio Uehara

**Affiliations:** Division of Clinical Nutrition, Faculty of Home Economics, Kyoritsu Women's University, 2-2-1 Hitotsubashi, Chiyoda, Tokyo 101-8437, Japan

## Abstract

We investigated the influence of maternal salt restriction during mating or gestation on birth rate and offspring growth in Dahl salt-sensitive rats (DS). DS were divided into 5 groups: DS fed a low-salt (0.3% NaCl, w/w) (DS-low) or high-salt (4% NaCl, w/w) diet (DS-high) during mating and DS-high or DS-low during gestation, and DS fed regular chow (0.75% NaCl, w/w) (DS-regular) throughout mating and gestation. During the unspecified periods, the rats were given regular chow. DS-low during mating delivered fewer infants than high-salt mothers (*P* < 0.05). The birth rate on regular chow was 87%. Six out of 11 DS-low rats during pregnancy produced pups while the rats fed a high-salt diet all delivered pups (*P* < 0.025). The pup survival rate was 67% for high-salt mothers during mating and 54% for mothers on a low-salt diet. The pup survival rate was 95% for mothers on a high-salt diet during pregnancy and 64% for mothers on a low-salt diet (*P* < 0.0001). Seven out of 8 DS-regular rats during mating delivered 59 neonates. However, 66% of the neonates survived. A low-salt diet during mating or pregnancy lowers birth rate and the neonates from low-salt mothers during pregnancy were more likely to die than those from high-salt mothers.

## 1. Introduction

For the last several decades, increased salt intake has been blamed for hypertension and related diseases, and a world-wide campaign on salt reduction commenced. Since the 1980s many investigators have reported serious problems in adult physiological and psychological health that are related to excessive salt intake. Despite such trends towards salt reduction and to our surprise, a recent meta-analysis including 25 previous studies on salt intake and cardiovascular events demonstrated that excessively low-salt intake is associated with an increase in cardiovascular events rather than benefits [1].

Some researchers reported that, particularly during pregnancy, low-salt intake is associated with adverse effects, that is, low birth weight and insulin resistance in offspring in adulthood [2–4]. Growth inhibition of astrocytes in the neonate brain, which occurs due to low-salt intake during pregnancy, is possibly involved in the dysfunction of the autonomic system in adulthood, thereby affecting hemodynamic or metabolic changes in adulthood [5]. Based on these data, there is a need to more carefully determine what the necessary salt requirements are and consider factors such as age or the stage of growth.

Accordingly, an increasing amount of evidence has suggested that fetal programming during gestation may play an important role in the development of metabolism-related disorders during adulthood [2, 6, 7]. Conceivably, genes are subjected to the influence of maternal food intake and lifestyle during gestation. During our investigation of the association between fetal programming and a postweaning salt appetite, we recognized that pregnant mothers on a low-salt diet appeared to deliver fewer babies than mothers on a high-salt diet (unpublished data).

To our knowledge, however, few studies have investigated the influence that maternal low-salt intake has on the process of fetal growth. There is a necessity to separately examine the influence of low-salt diet on fertilization and on fetal growth. Moreover, it is of interest to disclose the influence of low-salt intake in mothers on the growth of babies after weaning.

Thus, in the present study, we hypothesized that low-salt intake during mating (early pregnancy period) or pregnancy period influences fertilization and fetal growth and causes a decrease in the number of newborn pups. To test this hypothesis, we investigated the effects of a low-salt diet during mating or pregnancy on birth and growth rates of offspring in Dahl salt-sensitive (Dahl S) rats. Moreover, we examined the influence of low-salt intake on the growth of babies after weaning.

## 2. Materials and Methods

### 2.1. Study Design

We obtained 4-week-old male and female Dahl salt-sensitive (Dahl S) rats from Sankyo Laboratories, Inc., Tokyo, Japan. The rats were fed regular chow (0.75% NaCl, w/w) (CEL Rodent Diet CE-7, CLEA Japan, Inc., Tokyo, Japan). At 10 weeks of age, the rats were allowed to mate for 1 week (mating period (early pregnancy period)). Next, the mated female rats were placed in separate cages for the pregnancy period. Three weeks after mating, the mothers began delivering pups. We monitored the number of mothers that delivered pups, the number of pups delivered, and the growth of pups until weaning.

**Figure 1:**
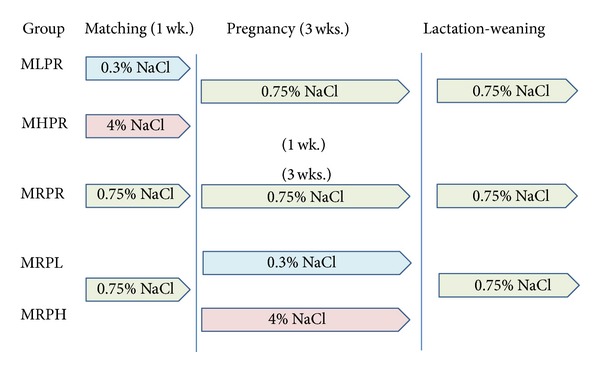
Protocol of the study. This graph shows each type of salt diet and the time period in which it was fed to the Dahl S rats. 0.3% NaCl, a low-salt (0.3% NaCl, w/w) diet; 0.75% NaCl, regular (0.75% NaCl, w/w) diet; and 4% NaCl, a high-salt (4% NaCl, w/w) diet.

Dahl S rats were divided into 5 groups (Figure 1): (1) Dahl S rats that were fed a low-salt (0.3% NaCl, w/w) diet (F2Dahl-0.3%, Oriental Yeast Co., Ltd., Tokyo, Japan) for the mating period, (2) Dahl S rats that were fed a high-salt (4% NaCl, w/w) diet (F2Dahl-4%, Oriental Yeast) for the mating period, (3) Dahl S rats that were fed a low-salt (0.3% NaCl diet, w/w) diet for the pregnancy period, (4) Dahl S rats that were fed a high-salt (4% NaCl, w/w) diet (F2Dahl-4%, Oriental Yeast) for the pregnancy period, and (5) Dahl S rats fed regular chow (0.75% NaCl, w/w) throughout the mating and pregnancy periods.

Dahl S rats were fed the regular (0.75% NaCl, w/w) chow during the periods unspecified. Water was available* ad libitum*.

### 2.2. Statistical Analysis

All statistical analyses were performed using STATISTICA software (StatSoft, Tulsa, OK). Values were expressed as means ± SD. Differences were assessed by *χ* ^2^ and one-way factorial analysis of variance (ANOVA) followed by post hoc least significant difference (LSD) test, Mann-Whitney *U* test, or Kaplan-Meier survival curve. *P* values less than 0.05 were considered statistically significant.

### 2.3. Guidelines for Handling Rats

We followed the guidelines for experimental animal handling, and our study was approved by the Animal Care Committee of the Kyoritsu Women's University. The experiment was conducted in accordance with the National Institutes of Health (NIH) guidelines.

## 3. Results

### 3.1. The Effects of a Low-Salt Diet on Neonate Birth Rates

Dahl S rats fed a low-salt diet during the mating (early pregnancy) period delivered less pups compared with those fed a high-salt diet (Table 1). In Dahl S rats that were fed regular chow, 6 out of 7 mothers delivered pups, and there were no differences in birth rates between the high-salt rats and the rats fed regular chow.

Similarly, Dahl S rats fed the low-salt diet during the pregnancy period delivered less pups compared with those fed the high-salt diet. There were no differences in the birth rates between Dahl S rats that were fed the high-salt rats and those on regular chow.

The number of babies delivered from one mother was equal among the 5 mother groups (Table 1).

### 3.2. The Effects of a Low-Salt Diet on the Growth of the Neonates

The pups that were born in each group and the offspring that survived during the lactation period are shown in Table 2. There were no differences in the number of pups that survived the lactation period between Dahl S rats fed the low- and high-salt diets during the mating (early pregnancy) period.

In contrast, only 64% pups from mothers that were fed the low-salt diet during the pregnancy period survived the lactation period, while the survival rate was 95% for the pups whose mothers were fed the high-salt diet (*χ* ^2^ = 19.88; *P* < 0.0001).

**Figure 2:**
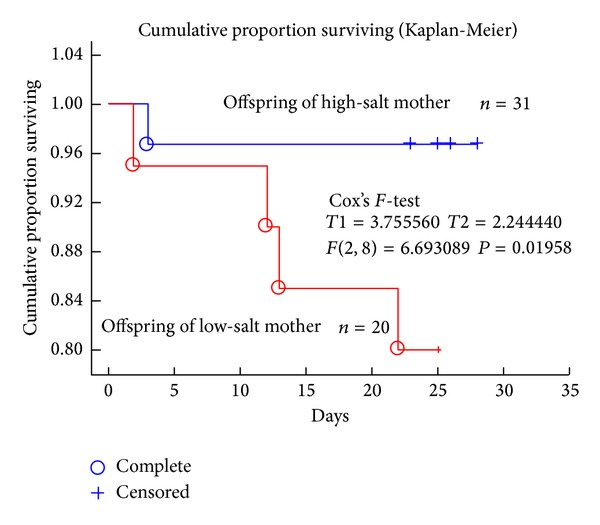
Cumulative proportion of offspring survival (Kaplan-Meier analysis). Survival prognosis was evaluated from their birth of the rat pups to weaning. Blue circles refer to the death (complete) of pups from high-salt intake mothers; red circles refer to the death (complete) of pups from a low-salt intake mothers. Plus (+), censor. The difference was analyzed by Cox's *F*-test (*T*1 = 3.755, *T*2 = 2.244, *F*(2, 8) = 6.6930, and *P* = 0.01958).

Using the data from the pups of mothers that were fed the low- and high-salt diets during the pregnancy period, we determined the Kaplan-Meier survival curve in order to increase our understanding of the time course before death (Figure 2). Some of the pups died immediately after birth and a substantial number of pups died during the lactation period.

We assessed pup growth during the lactation period (Table 3). Female pups born to mothers that were fed the low-salt diet tended to have lower body weights than pups born to mothers that were fed the high-salt diet. However, the difference was not significant. On the other hand, the body weights of the male pups from mothers that were fed the low-salt diet were equal to those of pups from mothers that were fed the high-salt diet.

## 4. Discussion

In the present study, we attempted to examine the role that salt intake has on the health of mothers and their children. We demonstrated that mothers on a low-salt (0.3% NaCl) diet during the mating pregnancy period produced lower number of pups than those on a high-salt (4% NaCl) diet. Since 7 out of 8 mothers that were fed regular chow throughout the mating and pregnancy periods produced babies, the low-birth rate of pups from mothers that were fed the low-salt diet during the mating period may be due to the failure to achieve fertilization. However, the mating season usually lasts 6-7 days in rats, and this means that time point of fertilization varied in mothers tested. The separation on the influence on fertilization and pregnancy process is technically difficult in the present experimental setting.

Similarly, the mothers that were fed the low-salt diet during the pregnancy period produced less pups than those that were fed the high-salt diet. As shown in mothers that were fed 0.75% NaCl throughout the mating and pregnancy periods, 7 out of 8 mothers produced pups, suggesting that the low-birth rate of pups from mothers that were fed the low-salt diet during the pregnancy period was due to a failure to maintain the pregnancy. In the present study, we were not able to conclude that fertilization is affected by the low-salt diet; however, it may be concluded that low-salt diet during the mating period and following pregnancy decreases birth rate.

In addition, we demonstrated that there were no differences in the number of babies born per pregnant mother among the experimental groups. These findings suggested that ovulation may not be affected by a low-salt diet.

The mechanism underlying why fewer pregnant mothers on a low-salt diet delivered pups is unclear. We speculate that low-salt intake enhanced the activity of the renin angiotensin system (RAS), and this may affect the intrauterine growth of pups. Moreover, some studies suggest that the overexpression of RAS transgenic mice results in a placenta-fetus imbalance and leads to preeclampsia [8–10]. In the present study, it is feasible to conclude that enhanced RAS activity following salt restriction led to a placenta and fetal imbalance, thereby decreasing the number of mothers that produced pups. Furthermore, recent studies have described the epigenetic mechanism for growth of the babies during pregnancy period [2, 4]. Events that are triggered after a low-salt challenge, that is, upregulation of the renin-angiotensin system, are believed to be involved in the epigenetic mechanism.

We demonstrated in the present study that rat pups born to mothers that were on a low-salt diet during a pregnancy period were more likely to die before weaning than those born to mothers that were fed a high-salt diet. However, it was also demonstrated that there were no differences in survival rate among the pups from mothers fed 0.75% NaCl or a low-salt diets during a pregnancy period. The 0.75% NaCl chow did not influence the birth rate of pups, but it lowered the survival rate of pups from mothers in comparison with pups from mothers fed a high-salt diet during a pregnancy period. This was demonstrated in Kaplan-Meier survival analysis. Since 95% of the pups from mothers on a high-salt diet during a pregnancy period survived until weaning, it was suggested that the regular (0.75% NaCl) diet was less likely to prevent the death of pups during a lactation period. The sensitivity of salt intake to benefits for mothers and pups differed.

In the present study we investigated Dahl S rats for the implications of mating or gestational salt intake in the birth and growth of the offspring. It is unclear whether salt sensitivity contributes to the relationship of mothers that were on a low-salt diet and their offspring. The future direction of research regarding salt intake will entail evaluating whether these findings are also true in other genetic rat strains of spontaneous hypertension.

We investigated the influence of salt intake on gestation and baby growth using Dahl S rats. Dahl S rats are a genetic model for salt-induced hypertension in humans [11, 12]. This strain develops hypertension with insulin resistance, depending on their salt intake. In addition, salt-induced hypertension is associated with target organ damage, that is, cerebral stroke, heart failure, and renal failure. These properties are similar to the conditions that develop in humans who have metabolic syndrome. There have been few studies on a public basis about salt sensitivity in Japan. However, it is believed that not less than 50% of Japanese people may be classified as salt sensitive. This figure is higher than that observed in Western countries [13, 14]. Based on the data, Dahl S rats are more appropriate for investigating the potential benefits or adverse effects of salt intake than the rat model for spontaneous hypertension (SHR).

The role of salt intake in the development of hypertension in humans has been the main theme over several decades, and this led to a world-wide campaign of salt reduction. However, the extent of salt reduction is controversial. Recently, a meta-analysis involving 25 studies reported that excessive salt intake is associated with an increased incidence of cardiovascular diseases [1]. Moreover, low-salt intake during gestation affects the development of fetuses and may contribute to hypertension or insulin resistance in their adulthood. In the present study, we provided evidence that the amount of salt intake for health benefits varies depending on each life stage. We may not extrapolate from the present results to humans. Investigation on humans is required.

In conclusion, low-salt intake during mating and pregnancy is associated with low-birth rates and high death rates among the surviving offspring. We urge additional investigations into the effects of paragestational salt reduction in humans as the level of salt intake that is required for optimal health varies depending on the subjects' age or stage of growth. Furthermore, the current finding provides evidence that supports the recommendation that the amount of salt consumed during pregnancy should be considered carefully based on data regarding the relationship between mothers and their offspring.

## Figures and Tables

**Table 1:** Number of mothers that delivered pups.

Group	Diet		Mother			Statistics		
	Mating	Pregnant	Total	Delivered	Babies/mother∗	*χ* ^2^	*P*	Versus
	(NaCl, w/w)	(NaCl, w/w)	(rats)	(rats)	(rats)			
MHPR	4.0%	0.75%	7	7	7.4 ± 2.3			
MLPR	0.3%	0.75%	7	4	8.7 ± 1.7	3.82	0.050	MHPR

MRPR	0.75%	0.75%	8	7	8.2 ± 1.4	0.94	0.332	MHPR
						1.76	0.184	MLPR
						1.45	0.228	MRPH
						2.33	0.127	MRPL

MRPH	0.75%	4.0%	11	11	10.2 ± 1.3			
MRPL	0.75%	0.3%	11	6	8.8 ± 3.0	6.87	0.011	MRPH

MHPR: mothers fed the high-salt diet during mating period and the regular chow during pregnancy period; MLPR: mothers fed the low-salt diet during mating period and the regular chow during pregnancy period; MRPR: mothers fed the regular chow during mating period and during pregnancy period; MRPH: mothers fed the regular chow during mating period and the high-salt diet pregnant period; MRPL: mothers fed the regular chow during mating period and the low-salt diet during pregnant period. Total: the total number of mothers; delivered: the number of mothers that gave birth to pups. The differences in delivered mothers were assessed by *χ* ^2^ test (df = 1). 0.75% NaCl (w/w) chow is usually used for maintaining rat colonies.

∗: number of rats delivered per mother.

**Table 2:** Mortality rates of the newborn pups until weaning.

Group	Diet (NaCl, w/w)		Rats born (mother∗)	Survival rate during lactation		∗∗Statistics		
	Matching	Pregnant		Alive (%)	Dead (%)	*χ* ^2^	*P*	Versus
MHPR	4%	0.75%	52 (7)	35 (67)	17 (33)			
MLPR	0.3%	0.75%	35 (4)	19 (54)	16 (46)	1.51	0.21	MHPR

MRPR	0.75%	0.75%	59 (7)	39 (66)	20 (34)	0.02	0.89	MHPR
						1.30	0.25	MLPR
						26.82	0.00	MRPH
						0.05	0.82	MRPL

MRPH	0.75%	4.0%	113 (11)	107 (95)	5 (5)			
MRPL	0.75%	0.3%	53 (6)	34 (64)	19 (36)	19.88	0.00	MRPH

MHPR: mothers fed the high-salt diet during mating period and the regular chow during pregnancy period; MLPR: mothers fed the low-salt diet during mating period and the regular chow during pregnancy period; MRPR: mothers fed the regular chow during mating period and during pregnant period; MRPH: mothers fed the regular chow during mating period and the high-salt diet pregnancy period; MRPL: mothers fed the regular chow during mating period and the low-salt diet during pregnancy period. Rats born: the total number of newborn pups; alive: the number of rats that were alive until weaning; dead: the number of rats that died before weaning. ∗: mothers that delivered babies. ∗∗The differences in alive pups were assessed by *χ* ^2^ test (df = 1). 0.75% NaCl (w/w) chow (regular chow) is usually used for maintaining rat colonies.

**Table 3:** Body weights of pups during lactation.

	Day 2	Day 9	Day 16	Day 23	Day 30
LS-male	7.0 ± 0.2	12.6 ± 1.3*	24.6 ± 1.7**	40.0 ± 2.0	69.5 ± 3.3
HS-male	7.1 ± 1.1	14.4 ± 2.0	26.9 ± 1.8	41.2 ± 2.2	73.4 ± 4.4

LS-female	6.8 ± 0.3	13.4 ± 0.9**	25.0 ± 1.4	42.2 ± 2.9	71.0 ± 3.0**
HS-female	7.3 ± 1.1	14.9 ± 1.9**	26.3 ± 2.8	43.0 ± 2.6	75.3 ± 6.0**

LS: offspring of Dahl S rats fed a low-salt diet during the pregnancy period; HS: offspring of Dahl S rats fed a high-salt diet during the pregnancy period. The differences were assessed by ANOVA and followed by post hoc analysis. *P < 0.1 and **P < 0.05 versus offspring of mothers fed the high-salt diet.

## Abbreviations

Dahl S rats: Dahl salt-sensitive rats
SHR: Spontaneously hypertensive rats
RAS: Renin angiotensin system
SD: Standard deviation
ANOVA: One-way factorial analysis of variance.
